# Proteomics-Based Identification of Interaction Partners of the Xenobiotic Detoxification Enzyme FMO3 Reveals Involvement in Urea Cycle

**DOI:** 10.3390/toxics10020060

**Published:** 2022-01-28

**Authors:** Zhao Yang, Paul M. Stemmer, Michael C. Petriello

**Affiliations:** 1 Institute of Environmental Health Sciences, Wayne State University, Detroit, MI 48202, USA; zhaoyang@med.wayne.edu (Z.Y.); pmstemmer@wayne.edu (P.M.S.); 2 Department of Pharmaceutical Sciences, College of Pharmacy, Wayne State University, Detroit, MI 48202, USA; 3 Department of Pharmacology, School of Medicine, Wayne State University, Detroit, MI 48202, USA

**Keywords:** FMO3, urea cycle, protein interactions, TMAO, proteomics

## Abstract

The hepatic xenobiotic metabolizing enzyme flavin-containing monooxygenase 3 (FMO3) has been implicated in the development of cardiometabolic disease primarily due to its enzymatic product trimethylamine-N oxide (TMAO), which has recently been shown to be associated with multiple chronic diseases, including kidney and coronary artery diseases. Although TMAO may have causative roles as a pro-inflammatory mediator, the possibility for roles in metabolic disease for FMO3, irrespective of TMAO formation, does exist. We hypothesized that FMO3 may interact with other proteins known to be involved in cardiometabolic diseases and that modulating the expression of FMO3 may impact on these interaction partners. Here, we combine a co-immunoprecipitation strategy coupled to unbiased proteomic workflow to report a novel protein:protein interaction network for FMO3. We identified 51 FMO3 protein interaction partners, and through gene ontology analysis, have identified urea cycle as an enriched pathway. Using mice deficient in FMO3 on two separate backgrounds, we validated and further investigated expressional and functional associations between FMO3 and the identified urea cycle genes. FMO3-deficient mice showed hepatic overexpression of carbamoylphosphate synthetase (CPS1), the rate-limiting gene of urea cycle, and increased hepatic urea levels, especially in mice of FVB (Friend leukemia virus B strain) background. Finally, overexpression of FMO3 in murine AML12 hepatocytes led to downregulation of CPS1. Although there is past literature linking TMAO to urea cycle, this is the first published work showing that FMO3 and CPS1 may directly interact, implicating a role for FMO3 in chronic kidney disease irrespective of TMAO formation.

## 1. Introduction

Flavin-containing monooxygenase 3 (FMO3) is an endoplasmic reticulum-tethered protein primarily expressed in the liver of adult humans [1], which is known to induce N- or S-oxygenation of numerous drug substrates [1] and has recently gained much attention for its role in the formation of proatherogenic trimethylamine-N oxide (TMAO). Flavin-containing monooxygenases are a major class of enzymes responsible for oxidizing multiple substrates, including many commonly administered amine- and sulfate-containing drugs, including Clozapine, Ranitidine, Tamoxifen, Atazanavir, Abacavir, and Lamivudine [1,2]. Distinguished from the major xenobiotic detoxification enzymes, cytochrome P450s, which require their accessory proteins (CYP reductases) to transfer electrons from NADPH, FMO3 directly accepts electrons from NADPH [1,3]. FMO3 expression is modulated by multiple transcription factors, including the aryl hydrocarbon receptor (AHR), farnesoid X receptor (FXR), and CCAAT/enhancer-binding protein beta (C/EBPbeta), as well as by steroid hormones, but inter-species differences are evident [4,5,6,7,8,9,10]. Although less inducible than CYP450s, induction of FMO3 and its metabolite TMAO have also been implicated in the cardiometabolic toxicity of environmental pollutants, including dioxin-like polychlorinated biphenyls (PCBs) [9].

FMO3’s association with metabolic disease has been thus far primarily mediated by its enzymatic product TMAO, which has been identified as a biomarker of cardiovascular disease, diabetes, and kidney disease [11]. In 2011, it was first described that TMAO could be formed in a gut-microbe-dependent manner from dietary sources including eggs, and that TMAO levels predicted stroke and heart attack risk in humans [12]. TMAO’s precursor, trimethylamine (TMA), is generated from dietary methylamines (e.g., choline, carnitine, betaine, and lecithin, abundantly found in diets rich in meat and dairy products [13]) by the intestinal microbiota. TMA is then oxidized to TMAO by hepatic flavin-containing monooxygenases, predominantly the FMO3 isoform [12]. We became interested in TMAO as a possible mechanism linking exposure to dioxin-like pollutants with cardiometabolic diseases, because it has been shown previously in mice that FMO3 expression could be induced by this class of persistent organic pollutants in an aryl hydrocarbon receptor-mediated fashion [9]. We reported that exposure to multiple dioxin-like pollutants increased FMO3 expression and activity and could lead to increased formation of TMAO [9]. In a separate study, we showed that TMAO levels were significantly positively associated with circulating dioxin-like pollutant concentrations in a highly exposed human cohort [14]. However, more work needs to be completed to identify if TMAO may be a causative mediator of inflammatory diseases or simply a biomarker of FMO3 expression/activity.

TMAO has been shown to alter inflammatory pathways including nuclear factor-kappa B signaling and inflammasome signaling [15,16,17], but not all reports support the hypothesis that increased TMAO is unhealthy. For example, fish and seafood are enriched with TMAO, but fish consumption, unlike other protein sources, reproducibly shows inverse or no associations with increased risk of mortality due to coronary artery disease [18,19,20,21,22]. TMAO has long been studied as an osmolyte known to protect the stability of proteins, especially in marine animals [23,24]. However, the impact of modulating TMAO levels on protein folding and protein:protein interactions in humans has not been thoroughly investigated. Some work has however been completed to associate circulating TMAO levels with gene expression changes. For example, a genome-wide association study discovered a single-nucleotide polymorphism within carbamoylphosphate synthetase (CPS1) (rs715 (T > C)), a gene encoding the rate-limiting enzyme of the urea cycle, was associated with plasma TMAO levels in females [25]. The urea cycle converts waste ammonia to urea, a chemical, which interestingly has opposite effects on protein stability compared with TMAO in that urea diminishes the stability of protein folding while TMAO counteracts this [23,24,26]. It is not well established what is driving the association between CPS1 and TMAO in humans.

Here, we hypothesized that FMO3, a primarily microsomal protein, can impact on cardiometabolic disease through protein/protein interactions with other important mediators of disease risk. Proteomic approach is as an appropriate sample preparation and analytical tool to analyze differentially expressed proteins [27]. In our study, we aim to analyze protein–protein interaction partners (PIPs) of FMO3 by co-immunoprecipitation techniques coupled with untargeted proteomics to further understand a larger regulatory network impacted on by FMO3. Through in vitro and in vivo experiments, we demonstrate that FMO3 interacts with multiple other proteins, many of which are related to the urea cycle and mitochondrial function. In vivo loss of FMO3 (and subsequent TMAO) via Crispr/Cas9 interestingly modulates the mRNA and protein expression of some of these interaction partners. We confirm one such FMO3 interaction with CPS1 and show that loss of FMO3 increases hepatic urea levels. These results provide evidence of a novel mechanism linking FMO3 expression/activity to multiple biochemical pathways and point to the importance of studying endogenous and xenobiotic factors that may impact on FMO3 protein levels.

## 2. Materials and Methods

### 2.1. Animal

FMO3 knockout mice on C57BL/6 and FVB (Friend leukemia virus B) background were developed using Crispr/cas9 technology and were a kind gift from Dr. Sudha Biddinger (Boston Children’s Hospital). Animals were housed on a 12 h/12 h light/dark cycle with ad libitum water and rodent food. Mice were provided with standard chow. At the age of 9–10 months, male and female mice were euthanized, and their tissues were perfused, immediately frozen, and stored in −80 °C until being used. All animal experiments were performed in accordance with the protocols approved by the Institutional Animal Care and Use Committee (IACUC) for Wayne State University. For the proteomics study, stored samples from our previous manuscript describing inducibility of FMO3 were used [9].

### 2.2. Cell Culture

AML12 cells were purchased from ATCC and cultured in DMEM/F12 media (ATCC) supplemented with 10% FBS, 10 µg/mL insulin, 5.5 µg/mL transferrin, 5 ng/mL selenium, and 40 ng/mL dexamethasone at 37 °C with 5% CO_2_. Expression vectors expressing mouse FMO3 with Myc-DDK-tag (Origene, Rockville, MD, USA) were transfected with Lipofectamine 2000 (Invitrogen) according to the manufacturer’s protocol. After 48 h post-transfection, cells were washed with phosphate-buffered saline (PBS) and lysed in respective buffers.

### 2.3. Co-IP

Mouse livers were lysed in Tris-buffered saline (TBS) containing 25 mM Tris base pH 7.4, 150 mM NaCl, 1% NP-40, 5% Glycerol, and protease inhibitor cocktail (Thermo, Waltham, MA, USA). Liver extracts were immunoprecipitated overnight at 4 °C with protein a/g agarose beads (Thermo) conjugated with 10 ug anti-FMO3 (abnova) after being pre-cleaned with the same beads.

Co-IP protein lysate was buffered with 40 mM triethylammonium bicarbonate (TEAB), then reduced with 5 mM dithiothreitol (DTT) and alkylated with 15 mM iodoacetamide (IAA) under standard conditions. Excess IAA was quenched with an additional 5 mM DTT. The samples were then digested overnight with sequencing-grade trypsin (Promega, Madison, WI, USA). Digests were cleaned up by strong cation exchange fractionation (Next Group, SEM-HIL-SCX tips) according to standard protocol.

### 2.4. Mass Spectrometry Analysis

The peptides were separated by reversed-phase chromatography (PepMap RSLC C18 column, Thermo Scientific, Waltham, MA, USA), followed by ionization with the Easy-Spray Source (Thermo Scientific), and introduced into a Fusion mass spectrometer (Thermo Scientific).

Abundant species were fragmented with collision-induced dissociation (CID). Data analysis was performed using Proteome Discoverer 2.4 (Thermo Scientific), which incorporated the Sequest algorithm (Thermo Fisher). The Uniprot_Mus_Compl_20181221 database was searched for mouse protein sequences, and a reverse decoy protein database was run simultaneously for false discovery rate (FDR) determination. The data files were loaded into Scaffold (Proteome Software, Portland, OR, USA) for distribution. Sequest was searched with a fragment ion mass tolerance of 0.6 Da and a parent ion tolerance of 10 PPM. Carbamidomethylation of cysteine was specified in Sequest as a fixed modification. Deamidation of asparagine, oxidation of methionine, and acetylation of the n-terminus were specified in Sequest as variable modifications.

### 2.5. Immunoblotting

Cells or tissues were homogenized in TBS. Protein samples were separated by 10% SDS-polyacrylamide gel and then transferred to a nitrocellulose membrane. For Western blot analysis, the antibodies were diluted according to manufacturer’s instructions. The antibodies are listed in the Table S2. The signal of the protein was captured by the ChemiDoc MP Imaging System (Biorad, Hercules, CA, USA). Quantification of the protein relative level has been conducted by ImageJ software (NIH, Bethesda, MD, USA).

### 2.6. Urea/Ammonia Assays

Hepatic and serum urea and ammonia levels were measured by commercial kits (MAK006 and AA0100, Sigma-Aldrich, Saint Louis, MO, USA). Liver tissues were homogenized in the urea assay buffer provided by the kit for urea assay, while livers were homogenized in the water for ammonia assay, followed by protein concentration measurement to normalize the samples. Serum samples were measured by the kits directly.

### 2.7. RT-qPCR

Total RNAs purified by Trizol reagent (Invitrogen) were reverse-transcribed to cDNA for quantifying with an Applied Biosystems QuantStudio 6 Flex Real-Time PCR System using SYBR Green (Applied Biosystems, Waltham, MA, USA). Samples were analyzed as duplicates, and expression levels were calculated with the manufacturer’s software using the ΔΔCt method. The PCR primers are described in Table S3.

### 2.8. Statistical Analysis

The sample number (*n*) of biological independent samples is indicated in the related results section. The data were analyzed by the unpaired Student’s *t*-test. Significant *p*-values are indicated by asterisks in the figures; *p*  <  0.05 (*), *p*  <  0.01 (**) were considered statistically significant. Statistical analyses were performed with GraphPad Prism 6 software.

## 3. Results

### 3.1. Hepatic FMO3 Interacts with Multiple Proteins Involved in Urea Cycle

To begin to investigate if FMO3 may have a physiological role irrespective of its enzymatic activity, we have identified interaction partners of FMO3 in livers of basal FMO3 expression as well as from livers of mice highly expressing FMO3 [9]. To induce FMO3 protein expression, we have used our established model of chemical inducibility by PCB-126 exposure, which works through the Aryl hydrocarbon receptor to induce FMO3 in mice (Figure S1A). We used an FMO3 antibody suitable for co-immunoprecipitation, and normal IgG IP was used as a control for each group (Figure S1B). After Co-IP, FMO3 interaction complexes were determined in an unbiased manner by chromatography–tandem mass spectrometry proteomics (LC–MS/MS). The FMO3 antibody chosen successfully enriched for FMO3 protein during the pull down as it was detected in 9 out of 10 FMO3 IP samples and was not detected in normal IgG IP samples (samples without FMO3 detected were excluded in further analyses). Overall, a total of 1196 proteins were detected in any of the samples, and 107 proteins exhibited more than 1.5-fold enrichment in the FMO3 IP samples (*n* = 9) compared with normal IgG control (*n* = 10); including FMO3, which had, on average, a 2.8-fold enrichment in the FMO3 IP group (Table S1). To identify a more conservative list of interaction partners, we then ruled out proteins detected in less than half of the nine FMO3 IP samples, leading to the list of 51 proteins reported in Table 1. In Figure S1C, we report a protein interactome map based on existing protein interactors (Gray nodes) in the BioGRID database (thebiogrid.org (last accessed on 26 January 2021)) of the 51 FMO3 PIPs. Among these possible FMO3 PIPs, the only protein that has been previously reported in a mouse study is FMO5 [28]. No other previous studies have reported FMO3 PIPs in mouse liver.

**Figure 1:**
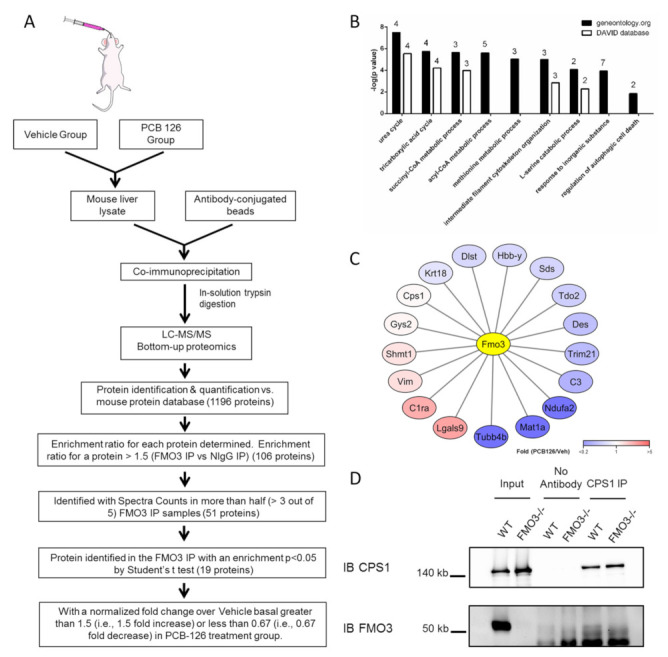
MS-based proteomic analysis of FMO3 protein–protein interaction partner network. (**A**) Schematic workflow of the antigen affinity purification strategy in combination with MS-based proteomics. Mouse liver samples from two groups, vehicle and PCB-126-treated, were immunoprecipitated by FMO3 antibody and normalized to normal IgG IP. (**B**) Graphical depiction of the top nine significantly enriched pathways for the FMO3 interaction partners identified in the Gene Ontology analysis. The number of proteins identified as FMO3 interaction partners in a particular pathway is indicated above the bar. The greater the −Log (*p*-value) value (i.e., the smaller *p*-value), the less likely a pathway is significantly enriched just by chance. (**C**) Fold change of the significantly enriched FMO3 protein–protein interaction partners induced by PCB-126 treatment. The proteins of interest (nodes) are colored according to their relative enrichment to PCB-126 treatment (red) or Vehicle (blue) and are ordered in a clockwise fashion relating to their relative enrichment. (**D**) Reverse immunoprecipitation of CPS1 shows its binding with FMO3. WT and FMO3−/− mouse livers were lysed, and Co-IP with CPS1 antibody was applied in the reverse Co-IP. Data show an FMO3 band in the CPS1 IP of the WT sample (lane 5) but not in the no-antibody control (lane 3).

**Figure 2:**
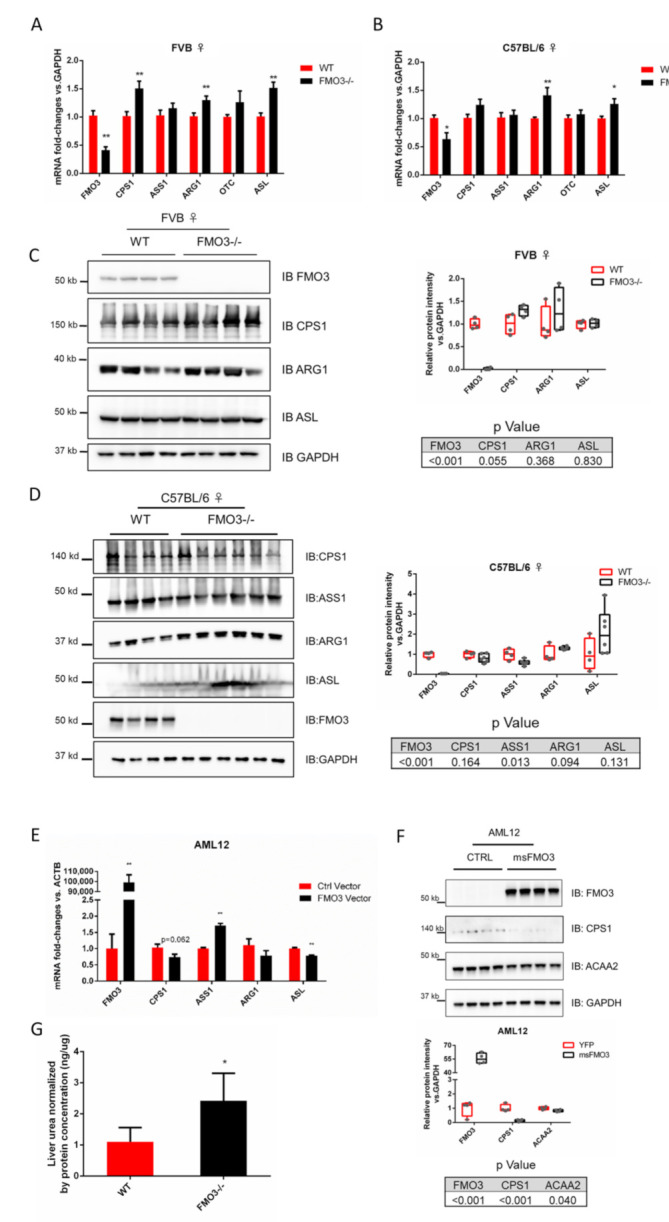
In vivo and in vitro data show expression and function of urea-cycle-associated FMO3 PIPs are regulated by the level of FMO3. The relative fold changes in transcription of five urea cycle genes were compared between the FVB (*n* = 7 (WT), 8 (FMO3−/−)) (**A**) or C57BL/6 (*n* = 7 (WT), 6 (FMO3−/−)) (**B**) female WT and FMO3−/− mouse livers. Red bars represent gene expression in the WT, while black bars represent FMO3−/− mouse results. * *p* ≤ 0.05; ** *p* ≤ 0.01. Protein expression level of FMO3 and its representative urea cycle proteins in the WT and FMO3−/− female mouse livers on the FVB (*n* = 4) (**C**) or C57BL/6 background (*n* = 4 (WT), 6 (FMO3−/−)) (**D**) are shown. Average fold change of the FMO3 PIPs shown in the right panel has been quantified and normalized by the housekeeping gene GAPDH. *p*-values of each gene are listed below the quantification figures. (**E**) mRNA of urea cycle genes in AML12 cells with/without FMO3 overexpression. *n* = 6. (**F**) Protein expression level of FMO3, CPS1 (as a representative of urea cycle gene), and ACAA2 (as another novel FMO3 PIP discovered in the previous proteomics) in the AML12 cells with/without FMO3 overexpression. Average fold change of the proteins shown in the below panel has been quantified and normalized by the housekeeping gene GAPDH. *p*-values of each gene are listed below the quantification figures. *n*= 4. (**G**) Hepatic urea levels normalized by total protein concentration in the FMO3−/− mice are significantly higher than those in the WT mice on the FVB background (*p* = 0.039). Quantification of protein expression shown in (**C**,**D**) 25 and 75 percentiles as box and whiskers plot, and all individual values were indicated as the points. Otherwise data are means ± SEM.

We were then curious to see if increasing FMO3 expression through chemical induction may impact on the binding of these PIPs. We compared spectral counts of each FMO3 PIP between vehicle and PCB-126-treated samples and identified 22 PIPs that were enriched in both groups (Table 1). We identified 19 proteins whose expression decreased by at least 1.5-fold due to FMO3 induction and 14 that were increased in PCB-126-treated samples (Figure S1C). In our BioGRID interactome map, we denote PIPs that were identified as enriched in vehicle samples (blue) or PCB-126 groups (red) and arranged in the inner circle. Reported mutual interaction partners of two or more novel FMO3 PIPs discovered in the current study were mapped in the outer circle (gray). In total, 80 proteins interact with at least two FMO3 PIPs. Our interaction map illustrates that FMO3 may directly or indirectly interact with the identified PIPs. Using the Gene Ontology (GO) resource (geneontology.org (accessed on 13 September 2021)) and DAVID bioinformatic database (david.ncifcrf.gov/ (last accessed on 13 September 2021)), we next identified biological processes that were significantly enriched for by the 51 FMO3 PIPs, and nine significant pathways were identified (Figure 1B), including urea cycle, intermediate filament, autophagic cell death, and response to inorganic substance (Figure 2B). Interestingly, multiple PIPs were identified that are localized to the mitochondria. The most significantly enriched biological process, urea cycle, was chosen as a target for subsequent functional analyses. Furthermore, we adopted more stringent criteria by selecting significantly changed FMO3 PIPs in the FMO3 IP group to obtain high-confidence results, and 17 proteins were obtained as a result, shown in Figure 1C.

### 3.2. FMO3 Interacts with CPS1 and Other Proteins of the Urea Cycle

To begin to validate the interaction of candidate FMO3 PIPs, we focus here on the pathway that exhibited the most significant enrichment, the urea cycle. This pathway included CPS1, ASS1, ARG1, and OTC. First, we isolated hepatic protein from female WT and FMO3−/− mice and completed a reverse Co-IP using a CPS1 antibody. In agreement with our unbiased proteomics results, FMO3 was detected by the reverse Co-IP in the wildtype (WT) but not in the FMO3 knockout (FMO3−/−) group (Figure 1D). Moreover, the FMO3 signal in the CPS1 IP group was stronger than in the no-antibody control (Figure 1D). This reverse Co-IP result helps to validate the interaction between FMO3 and CPS1, but strength of the interaction appears weak. Next, we were curious to see if the expression of our identified PIPs changed in mice that lacked FMO3. We focused on proteins highly enriched by FMO3 Co-IP, and we selected representative genes from the biological process/cellular component categories for further study. To accomplish this, we analyzed expression differences of genes of interest in mice of two distinct genetic backgrounds: C57BL/6J and FVB, and when possible, in both sexes (FMO3 shows strong sexual dimorphism).

At the mRNA level, a distinguishable expression pattern was observed in the female FMO3−/− mice in both backgrounds, especially for the urea-cycle-related genes (UCGs) (Figure S2A,B and Table 2). As a representative of UCGs, *Cps1* was induced 1.23-fold (*p* = 0.070) and 1.48-fold (*p* = 0.005) due to loss of FMO3 in mice of C57BL/6 and FVB background, respectively (Figure 2A,B). The expression of most other UCGs examined also showed increased expression due to loss of FMO3 (1.05~1.41-fold, Table 2). *Arg1* gene expression was significantly increased in FMO3−/− mice of the C57BL/6 (1.41-fold, *p* = 0.008) and FVB (1.28-fold, *p* = 0.008) background. Similarly, expression of *Glul*, a major enzyme of ammonia [29], was significantly increased in the FVB FMO3−/− mice (1.55-fold, *p* = 0.028), but not in the C57BL/6 background (1.05-fold, *p* = 0.594). Overall, FMO3 deficiency had a stronger impact on increasing expression of UCGs in the FVB compared with C57BL/6 background. One of the five core UCGs, ASL, did not pass the unbiased proteomic screening as a potential FMO3 PIP, but we included it for completeness and observed a significant increase in the mice of both backgrounds (C57BL/6: 1.26-fold; *p* = 0.018 and FVB: 1.50-fold, *p* = 0.001) (Figure 2A,B).

We also examined the expression level of PIPs not identified as UCGs in female FMO3−/− mice of both backgrounds. The expression of a type III intermediate filament protein, vimentin, was significantly increased in FMO3-deficient mice from the C57BL/6 (1.52-fold, *p* = 0.027) and FVB (1.53-fold, *p* = 0.034) backgrounds, respectively. Other genes, including *Mvp*, *Phb2*, *Slc25a3*, and *Dlst*, have shown significant induction due to loss of FMO3 in the C57BL/6 background but not in the FVB counterparts. For example, mitochondrial enzyme dihydrolipoamide S-succinyltransferase (*Dlst*) showed significantly increased expression in the C57BL/6 (1.34-fold, *p* = 0.035) but not in the FVB (1.15-fold, *p* = 0.119) female mice (Figure S2A,B and Table 2). On the contrary, expression of keratin18 was decreased due to loss of FMO3 in mice of the C57BL/6 (0.74-fold, *p* = 0.014) background, but not in mice of the FVB background.

Interestingly, many of these changes were not observed or were the opposite in male mice who normally contain very low basal levels of FMO3 [4]. Expression of Cps1 in the male C57BL/6 mice lacking FMO3 showed a decreasing trend (0.79-fold, *p* = 0.056), similar to the trend of change in the expression of vimentin (0.61-fold, *p* = 0.063) (Figure S2C). On the other hand, the only two significantly changed genes in the C57BL/6 male FMO3−/− mice analyzed by RT-qPCR, complement *C3* (0.61-fold, *p* = 0.007) and serine hydroxymethyltransferase 1 (*Shmt1*) (0.75-fold, *p* = 0.006), showed insignificant trends in their female counterparts (Figure S2C).

To determine if changes in mRNA expression were mirrored at the protein level, we completed western blotting of livers from female WT and FMO3−/− mice. In FVB FMO3−/− mice, CPS1 showed similar trends (1.30-fold, *p* = 0.055), which is consistent with the mRNA level, but other urea cycle proteins, ARG1 and ASL, showed no differences (Figure 2C). In FVB mice, protein level changes of the non UCGs also showed minimal expression level differences, and the only protein that showed a significant change was the mitochondrial carrier SLC25A3 (1.25-fold, *p* = 0.041) (Figure S2E). However, protein-level changes due to loss of FMO3 were not conserved in mice of different genetic backgrounds. In FMO3-deficient mice on the C57/BL6 background, expression of urea cycle proteins CPS1, ARG1, and ASL showed no significant change (Figure 2D), and ASS1 expression was significantly decreased in FMO3−/− mice (0.59-fold, *p* = 0.013). VIM (2.20-fold, *p* = 0.022) and KRT18 (0.06-fold, *p* = 0.003) showed significant changes in line with their changes at the mRNA level. Taken together, these results suggest that loss of FMO3 can have an impact on the expression level of FMO3 PIPs through unknown mechanisms.

Finally, to further validate some of our observations, we overexpressed murine FMO3 in a cell line that normally has low FMO3: AML12 hepatocytes. Strong overexpression of FMO3 led to decreased mRNA expression of the majority of urea cycle/ammonia-related genes, including *Cps1* (0.72-fold, *p* = 0.062), *Asl* (0.78-fold, *p* < 0.001), and *Glul* (0.71-fold, *p* < 0.001). The only exception was *Ass1*, which should significant induction (1.70-fold, *p* < 0.001). Multiple other genes showed similar trends that would have been predicted based on the studies using FMO3−/− mice. *Slc25a3* (0.80-fold, *p* < 0.001), *Acaa2* (0.69-fold, *p* < 0.001), and *C3* (0.75-fold, *p* = 0.036) all showed reduced expression due to FMO3 overexpression. Expression of *Vim* increased due to FMO3 overexpression (1.26-fold, *p* = 0.001), which would not have been predicted based on the in vivo results. We have validated the expression of two genes, CPS1 (0.14-fold, *p* < 0.001) and ACAA2 (0.83-fold, *p* = 0.040), at the protein level, and we observed consistent significant results (Figure S1D and Table 2)

### 3.3. Modulation of FMO3 Expression Impacts Hepatic Urea Levels

Among the biological processes in the GO analysis identified in our LC-MS/MS data, we focused on proteins of the urea cycle because their involvement in liver-associated systemic dysfunction such as hepatic encephalopathy has endowed them essential roles in liver function. We collected mouse serum and liver lysate prepared as in the manufacturer’s instructions, and measured ammonia and urea levels in these samples from female FVB WT and FMO3−/− mice (*n* = 4). As the result, average hepatic urea level in the FMO3−/− mice was 2.42 ng/ug protein, which was significantly increased compared with the level in the WT mice, which was 1.10 ng/ug protein (2.20-fold, *p* = 0.039), as shown in Figure 2D. Meanwhile, average hepatic ammonia level in the FMO3−/− mice increased from 0.16 to 0.25 ug/mg compared with WT mice, but this trend was not significant (1.56-fold, *p* = 0.058) (Figure S2G). However, both urea and ammonia levels showed no significant change in mouse serum between the two groups (*p* = 0.55 and 0.76, respectively) (Figure S2H,I). This result indicates that FMO3 deficiency leads to hepatic accumulation of urea. Taken together, the interaction between FMO3 and UCGs such as CPS1 affects not only the expression of the UCGs but also urea metabolism in the liver.

## 4. Discussion

Here, we identify a list of proteins that may directly or indirectly interact with FMO3, a xenobiotic detoxification enzyme that has been implicated as an important regulator of cardiometabolic disease. In addition to providing evidence for an understudied protein:protein interaction network, we show that modulation of FMO3 protein expression can impact on the mRNA and protein levels of many of these PIPs. Modulation of FMO3 impacts specifically on multiple genes related to the urea cycle, and we show that FMO3 deficiency leads to induction of hepatic *Cps1*, the rate-limiting enzyme of the urea cycle, as well as a subsequent increase in hepatic urea levels.

As with other FMO members, FMO3 is an endoplasmic reticulum-tethered protein and is majorly expressed in the liver of adult humans [1]. The FMO3 gene contains nine exons, and exon 1 is noncoding [30]. Multiple motifs have been identified throughout the FMO3 protein, including FAD-binding motif (GXGXXG within the region 3–26), NAPDH-binding motif (GXGXXG within the region 186–213), and FMO signature (xGxxxHxxxF/Y) and identifying (FxGxxxHxxxF) motifs [31,32,33]. In the yeast FMO protein, Asn91 (Asn61 in human FMO3) was believed to be the only amino acid involved in enzymatic activity of its substrates [3,34] until recently when new active sites involved in enzymatic activity have been discovered [35]. Furthermore, studies using point mutations have elucidated amino acids critical for its stability and metabolic activity, including Glu158, Val257 and Glu308 [36,37,38].

In addition to its best-known substrate trimethylamine, FMO3 induces N- or S-oxygenation of numerous drug substrates, reviewed in [1]. Most FMO3 substrates contain tertiary amine or sulfide in their structure. Distinguished from the major xenobiotic detoxification enzymes, CYP450s, which require their accessory proteins (CYP reductases) to transfer electrons from NADPH, FMO3 directly accepts electrons from NADPH [1,3]. The level and activity of FMO3 has been associated with metabolic diseases and disorders. The FMO3 metabolite TMAO has been identified as a cardiovascular disease marker in multiple studies [39,40]. The mRNA level of FMO3 has been to increase in male but decrease in female db/db type II diabetic mice compared with controls (db/-) [41]. Schugar and her colleagues discovered that FMO3 mRNA expression in men is positively correlated with obesity, and FMO3 knockout protects mice against high-fat-diet-induced obesity [42].

Herein, we used multiple means of modulating FMO3 protein expression to study impacts on possible protein:protein interaction partners. Expression of FMO3 is inducible by environmental factors such as dioxin-like PCBs. Studies published previously in our lab have shown a coplanar PCB called PCB-126 can induce greater than 100-fold overexpression of FMO3 in the male mouse [9,43]. Therefore, relatively lower expressions of FMO3 in the male mice and FMO3 inducer PCB-126 have been utilized in the proteomic experiment. In this study, we observed strong induction of FMO3 as well as increases in the FMO3 enzymatic product TMAO due to PCB exposure. Here, we used this model of FMO3 inducibility to identify PIPs in an unbiased means using a Co-IP-coupled proteomics-based strategy. We expected that most PIPs identified would show increased abundance in the PCB-126, FMO3 antibody group compared to Vehicle, FMO3 antibody group, but only 14 out of 51 FMO3 PIPs had at least a 1.5-fold increase induced by PCB-126 treatment (Table 1). Although it is not clear why more PIPs were not enriched due to chemically induced FMO3, it is possible that the supraphysiological increase of FMO3 disrupted the kinetic balance of protein interactions. In addition, we have shown that modulation of FMO3 can impact on the expression of some of these PIPs, which may also have played a role in this unexpected result.

Reverse Co-IP data showed that CPS1 interacts with FMO3, but this interaction is rather weak (Figure 1D). Weak and transient protein:protein interaction has been understudied, but it is critical for regulating cellular activity due to its high flexibility and sensitivity to environment [44,45]. Another possibility is that a protein interaction complex was formed including FMO3 and some of its novel interaction partners discovered in the study. As a result, FMO3 may be involved in an unknown regulatory protein complex.

Other mitochondrial-localized proteins have been identified as FMO3 interaction partners, which was surprising based on the known localization of FMO3 to the endoplasmic reticulum (ER). It leads to the possibility that FMO3 may play a role in the contact and material exchange between ER and mitochondria [46,47]. Multiple mammalian protein pairs have been identified to link the ER and mitochondria at the ER–mitochondria contact sites (ERMCSs), including MFN1/2, VDAC1/HSPA9/IP3R, VAPB/RMDN3, and BCAP31/FIS1 [48,49,50]. The ER–mitochondria contact facilitates exchange of cellular materials, especially lipids and calcium, and plays critical roles in mitochondrial division [47,48]. Validation of these hypothetical functions of FMO3 requires extensive future evidence.

Protein interaction partners of FMO3 published in previous articles can be used as quality controls; however, such studies that report on these types of interactions are rare. Only three interaction partners of FMO3, i.e., FMO5, FMO2, and DOCK3, have been identified in the BioGRID database (thebiogrid.org/ (accessed on 13 September 2021)), which originated from one research publication. In this publication, Pourhaghighi et al. [28] discovered a protein interactome in mouse brain, identifying over 1000 protein complexes in a brain interactome map, including three FMO3 protein complex pairs. Among these FMO3 partners, FMO5 and FMO2 have been identified in our proteomic dataset, but FMO2 has been ruled out since it showed spectra counts in less than half of the FMO3 IP-ed samples (4 out of 10). DOCK3 is highly expressed in the brain but not in the liver, according to the GTEx database (gtexportal.org (accessed on 13 September 2021)), which could explain its absence in our proteomic data.

Our data indicate direct or indirect interaction between FMO3 and FMO5 at the protein level. Interestingly, RT-qPCR and western blotting data from a published thesis indicated that mRNA levels of both FMO3 and FMO5 increase during postnatal development (1–198 days) in the human liver. Protein levels of the two FMOs follow the same increasing trend except for during the early postnatal phase (1–31 days) [51]. Zhang et al. analyzed tissue-specific expression of FMOs [52] and reported that FMO3 and FMO5 have the highest expression in the adult liver, and lower expression in the kidney and lung. Data in a dissertation showed that mRNA expression of other FMOs was not changed in the FMO3 knockout mouse, regardless of sex [53].

The expression of FMO3 can be regulated by intrinsic and environmental factors. Studies a decade earlier have discovered transcriptional regulation of FMO3 expression in the liver. For instance, supplementation with the bile acid, cholic acid, increased FMO3 expression in both sexes of mice fed with high-fat, cholesterol-containing diet. Further analysis discovered that an agonist of a bile acid-inducible nuclear receptor, farnesoid X receptor (FXR), increased FMO3 expression in the WT but not in the FXR–/– mice, revealing that FXR positively regulates the hepatic level of FMO3 [4]. The other transcription factor that regulates metabolism, CEBPB, has been reported to upregulate expression of FMO3, through protein-DNA binding to C/EBP element within FMO3 promoter domain [54]. Interestingly, aryl hydrocarbon receptor (AHR) has been shown to induce expression of FMOs in response to dioxin-like compounds such as TCDD and PCB-126 [9,55]. For example, TCDD-induced overexpression of FMO3 has not been observed in AHR−/− mice [56]. Although liver-elevated expression of FMO3 is universal in a large variety of species [5,6,7,8], sexual dimorphic expression of hepatic FMO3 is divergent among species. Expression of FMO3 shows a dramatic sexual dimorphism in the mouse, but studies fail to observe sexual dimorphism of FMO3 protein expression and enzymatic activity in human [8,57,58]. Results have shown more recently that at the mRNA level, females have a significantly higher expression of hepatic FMO3, though fold change between sexes in human (1–3 fold) is not comparable with the fold changes in the thousands observed in mouse [4,59]. Level of FMO3 in gonadectomized mice indicated that testosterone is the major inhibitor and 17b-estradiol is the minor promoter of FMO3 expression [60].

The AML12 cell line has been utilized previously in other in vitro studies of FMO3 overexpression. AML12 cell is a mouse hepatic cell line that expresses human TGFα cDNA controlled by mouse metallothionein 1 promoter [61]. The cell line has been widely used for studying hepatic lipid metabolism and liver injury largely due to its similarity in function and intracellular signaling compared to primary cells [62]. We overexpressed FMO3 in AML12 cells by transfection. As a result, mRNA level of urea cycle gene *Asl* was significantly decreased in the FMO3 overexpression group (Figure 2E). Protein level of CPS1 also showed a significant decrease, which is consistent with the trend shown at the mRNA level (Figure 2F). Interestingly, mRNA of *Ass1* was significantly increased in the overexpression group; the opposite trend was observed for other urea cycle genes (Figure 2E). Other studies have used a similar approach to identify impacts of FMO3 overexpression on biochemical processes using genetic modification or chemical treatment, and have discovered possible roles for FMO3 in metabolic processes other than urea cycle. Shih et al. discovered that in the FMO3-overexpressed Hep3B cells, mRNA levels of gluconeogenesis genes *GPT*, *G6PC1*, and *PCK1* have shown a significant increase in the cells with adenoviral overexpressed FMO3 [63]. On the protein level, the autophagic pathway was inhibited in the aged male FMO3 overexpressed mouse liver [64]. Moreover, adenoviral overexpressed FMO3 increased not only hepatic expression of inflammation and ER stress genes (CD68, ADGRE1, and ATF3) but also the targets of cholesterol-regulating transcription factor LXR (ABCA1 and LPCAT3), modifying cholesterol balance in the mice [65].

FMO3 has gained much attention within the past 10 years primarily because one of its enzymatic products, trimethylamine N-oxide (TMAO), has been identified as a biomarker of coronary artery disease, diabetes, and chronic kidney disease. There is evidence to support a causative role for TMAO in some of these pathological states. An elevated TMAO level has been reported to promote activation of nuclear factor-κB (NFKB) signaling, which may induce accumulation of low-density lipoprotein particles in the artery wall [15]. Another recent study has shown that TMAO can also activate the NLRP3 inflammasome, leading to increased vascular calcification [66]. Moreover, TMAO itself can amplify platelet hyper-reactivity and thus induces increased thrombosis risks [67]. Our collaborative group recently showed that TMAO can bind and activate the PKR-like ER kinase (PERK) pathway. TMAO binds and induces phosphorylation of PERK in the hepatocytes [68]. DDIT3 (CHOP), a downstream effector of PERK pathway, has been reported to associate with atherosclerotic lesion progression [69]. Interestingly, PERK has been reported to activate NFKB signaling [70] or NLRP3 inflammasome [71], which indicates a potential inflammatory regulation network of TMAO-induced CVDs. It is established that FMO3−/− mice produce negligible levels of TMAO, and thus it is possible that loss of TMAO in our studies may have critical impacts on the expression of some of our PIPs.

**Figure 3:**
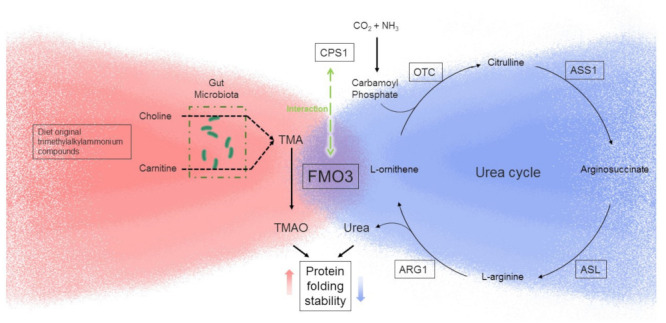
FMO3 interacts with CPS1 and may impact on protein stability through modulating urea/TMAO ratio. The canonical enzymatic activity of TMAO production by FMO3 is depicted on the left, and the canonical urea cycle is shown on the right part. Trimethylalkylammonium compounds found in diet can be metabolized to trimethylamine (TMA) by gut microbiota. Hepatic FMO3 catalyzes oxidation of TMA and results in production of TMAO. We provide evidence that FMO3 interacts with and may impact expression of CPS1, the rate-limiting enzyme of urea cycle, leading to modulation of hepatic urea. TMAO is known to increase while urea is known to decrease protein folding stability.

One of the highlighted pathways in which FMO3 interaction partners have participated, urea cycle signaling, has shown expressional change of its rate-limiting enzyme CPS1 as well as functional changes (Figure 3). Urea cycle is the major contributor to excrete excess metabolism-produced ammonia, which is generally localized to the periportal hepatocytes [29]. Urea cycle enzymes catalyze excess ammonia to lower toxic metabolite urea for excretion to prevent hyperammonemia. Major enzymes involved in this process include carbamoylphosphate synthetase (CPS1), ornithine transcarbamylase (OTC), argininosuccinate synthetase (ASS), argininosuccinate lyase (ASL), and arginase (ARG) [72]. Urea cycle gene CPS1 has been associated with TMAO in previous studies. A GWAS study found that CPS1 localized SNP rs715 (T > C) is associated with plasma TMAO level in females, which increases the risks of atherosclerosis [25]. Interestingly, USCS genome browser (http://genome.ucsc.edu (last accessed on 26 January 2021)) indicated that rs715 locus is conserved in most mammalian species, excluding partial rodents such as house mice. It suggests that rs715 is not the sole factor involved in the regulation of TMAO level by CPS1, but its physical interaction with FMO3 may also play a role.

Urea cycle converts waste ammonia to urea, a chemical that has opposite effects on protein stability against TMAO. Urea undermines stability of protein folding, while TMAO counteracts and rescues the destabilizing effect of urea [23,24]. The chemical mechanism of urea–TMAO interaction and how this interaction affects the stability of protein folding has been debated for decades [23,24]. Therefore, whether urea–TMAO interaction plays a role in expression changes of FMO3 interaction partners is an intriguing question to be answered. In our FMO3−/− mouse model, animals were exposed to an inherent TMAO-deficient environment, which may gradually alter intracellular interaction and expression network. Therefore, an acute FMO3-deficient model is required for future exploration. Previous studies have applied antisense oligonucleotides (ASO) to knockdown FMO3 in vivo [59,63,65,73]. Diana et al. discovered that ASO-mediated knockdown of FMO3 significantly reduced plasma lipid level, including triglyceride, VLDL, and LDL [63]. Their study also indicated a decreasing plasma urea level, but the hepatic urea level was not mentioned. However, a recent study [73] has identified an off-target effect of the FMO3 ASO on multiple metabolic genes. It discovered phenotypic inconsistency in metabolic regulation, including in plasma triglyceride levels and diabetes-associated atherosclerosis. Our data showed in the 9–10-month-old female FVB mouse model, urea level was significantly increased in the liver but not in the serum (Figure 2G,I). The decreasing plasma urea was observed in the FMO3 ASO-injected LDLR knockout mice [63], which may also be explained by the off-target effect of ASO. However, inconsistent observation of hepatic and serum urea levels in the FVB mice remains unexplained. We hypothesized that the plasma-membrane-tethered urea transporters SLC14A1 and SLC14A2 are unchanged in the FMO3−/− mice; consequently, hepatic urea export is not determined by intracellular urea level. The other explanation is that renal urea excretion will be upregulated to meet high level of urea in the FMO3−/− mice. Heavier renal burden of urea excretion induced by FMO3-deficiency-mediated UCG dysregulation may be associated with predisposition of chronic kidney disease in the FVB mice [74,75]. Collectively, a conditional FMO3 knockout mouse model is the optimal option to analyze effects of FMO3 deficiency on its interaction partners with minimal interference by constitutive deficiency of TMAO.

Various transcription factors have been reported to be involved in hepatic transcriptional regulation of CPS1, contributing to a complex regulatory network of urea cycle. C/EBPα and C/EBPβ bind to C/EBP recognition elements located in CPS1 promoter region and initiate Cps1 transcription under different physiological conditions [10,76,77]. Glucocorticoid receptor (GR) enhances transcription of Cps1 in a C/EBP- and HNF3-dependent manner [78]. More recent studies have identified that other transcription factors such as CREB and FOXA are involved in transcription of Cps1 [79,80]. Singh et al. recently published a preprint study that discovered hypoxia-inducible factors (HIFs) may upregulate transcription of Cps1 [81]. Interestingly, some transcription factors, such as JUN, inhibit transcription of Cps1 when binding to the cis-element [82]. Tumor suppressor p53 has been reported to inhibit transcription of UCGs, including CPS1 [83]. In addition, upregulating DNA methylation is another mechanism to inhibit transcription of Cps1 [84], and post-translational modifications (PTMs) change the activity of CPS1 at the protein level [85,86,87]. Currently, we do not have data to show whether FMO3 regulates transcription of Cps1 or protein stability of it. Furthermore, it has been reported that NFKB, a transcription factor that can be activated by TMAO [15], induced expression of CPS1 in bladder tumor cell lines [88]. As the result, whether TMAO is involved in FMO3-regulated expression of Cps1 remains unclear. Interestingly, data suggested the relationship between FMO3 and ammonia. A proteomic study on broiler chicken liver indicated that expression of FMO3 had a 1.99-fold increase in ammonia-treated samples compared with the controls [89]. FMO3 has been shown to oxidize ammonia in vitro [90]. In our data, loss of FMO3 led to an increase of hepatic ammonia (*p* = 0.058). Taken together, to understand the mechanisms by which FMO3 regulates the urea cycle, especially the expression of CPS1, requires further study.

Finally, we observed some differential effects due to loss of FMO3 in our two distinct mouse backgrounds. Although we do not know conclusively why loss of FMO3 seemingly had a stronger impact on PIPs in mice of the FVB background, it is possible these discrepancies were due to genetic or other endogenous factors. Previous literature has reported differences in gene expression [91,92,93] and response to chemicals [94] between the two mouse backgrounds. It has been identified that the background and sex-specific change in Fmo3 expression and TMAO levels react to dietary cholic acid [4]. Our data showed multiple UCGs had higher fold changes and lower *p*-values on the FVB background FMO3−/− mice, while other FMO3 PIPs showed less significance when they compared with mice on C57BL/6 background (Table 2). Given that elevation of the FMO3 metabolite TMAO has been associated with severity of renal dysfunction clinically [95,96], and FVB mice inherit a predisposition to nephropathy compared to other strains, especially the C57BL/6 [74,75], our data reveal a possibility that FMO3 can exacerbate nephropathy through regulating the expression of urea cycle genes in the liver, which requires further evidence. The composition of gut microbiota is affected by exogenous and endogenous factors and is an indicator of health and disease [97]. Through enterohepatic circulation, the balance between ammonia and urea changes and causes the flourish of related gut flora, including urease-containing bacteria (UCB), ammonia-oxidizing bacteria (AOB), and ammonia-producing bacteria [98,99,100,101]. Our unpublished data suggested a distinguished gut microbiome in which a group of UCB is significantly altered in the FMO3−/− mice. Whether this change is associated with the abnormal hepatic urea and ammonia observed in the FMO3−/− mice will be determined in the future studies.

## 5. Conclusion and Future Direction

In conclusion, here we discovered novel protein:protein interaction partners of FMO3 and report the involvement of FMO3 in regulating urea cycle through its interaction partners. Our study provides evidence for important roles of FMO3 in biochemical signaling beyond its production of TMAO, but more work is still needed to determine if upregulation of FMO3 due to chemical or nonchemical stressors is important in overall cardiometabolic disease risk. In the future, multiple directions can be pursued, including analysis of subcellular effects of FMO3–CPS1 interaction and systematic investigation of how modulation of FMO3 expression may impact on protein stability through modulation of urea/TMAO ratios.

## Figures and Tables

**Table 1:** Co-IP enrichment fold and PCB-126-treatment-induced changes of 51 FMO3 PIPs.

Gene Name	FMO3 IP vs. NIgG IP						Fold Change Norm. by NIgG IP (PCB Treated/Vehicle)	Half Sample Size Criteria
	General (*n* = 9)		Vehicle (*n* = 5)		PCB-126 Treated (*n* = 4)			
	Enrichment Fold	Enrichment *p*-Value	Enrichment Fold	Enrichment *p*-Value	Enrichment Fold	Enrichment *p*-Value		
*CPS1*	1.7	0.014	1.7	0.186	1.7	0.037	1.2	Passed in both groups
*ASS1*	0.8	0.786	0.5	0.392	1.7	0.424	4.0	
*ARG1*	1.8	0.170	2.5	0.308	1.4	0.373	0.7	
*ACAA2*	1.5	0.240	1.5	0.471	1.5	0.422	1.2	
*C3*	13.8	0.000	19.0	0.000	9.1	0.016	0.6	
*CYP1A2*	1.4	0.553	7.3	0.138	1.2	0.568	0.2	
*DES*	3.1	0.022	4.1	0.064	2.2	0.264	0.7	
*DLST*	9.5	0.000	11.0	0.001	7.0	0.015	0.8	
*FMO5*	1.8	0.330	2.4	0.394	1.0	0.804	0.5	
*GSTM1*	1.3	0.497	1.7	0.479	1.1	0.654	0.8	
*GYS2*	1.8	0.000	1.6	0.014	2.0	0.015	1.3	
*HBB-Y*	3.4	0.000	3.8	0.007	3.0	0.016	0.8	
*KRT18*	5.2	0.020	5.9	0.126	4.1	0.037	0.9	
*KRT8*	2.1	0.102	1.6	0.386	2.7	0.204	2.1	
*MRI1*	6.5	0.002	34.0	0.041	3.4	0.042	0.1	
*MVP*	1.5	0.358	1.3	0.716	1.7	0.414	1.6	
*RPL13*	0.9	0.984	3.0	0.282	0.3	0.085	0.1	
*SHMT1*	2.9	0.049	2.4	0.276	3.4	0.134	1.8	
*SLC25A3*	1.4	0.392	0.9	0.880	2.0	0.278	2.8	
*TDO2*	13.0	0.004	15.0	0.019	9.0	0.079	0.8	
*TUBB4B*	4.4	0.036	12.0	0.100	2.5	0.259	0.3	
*VIM*	5.8	0.005	5.0	0.137	7.5	0.000	1.9	
*ATP5PD*	1.5	0.427	3.3	0.123	0.7	0.926	0.3	Passed in the Vehicle group
*C1QB*	1.9	0.156	2.0	0.351	1.8	0.368	1.1	
*C1RA*	12.0	0.021	1.6	0.014	4.0	0.407	3.1	
*ETFA*	1.0	0.825	1.5	0.562	0.5	0.543	0.4	
*FTCD*	1.4	0.640	2.9	0.443	0.3	0.382	0.1	
*GLUL*	2.9	0.106	6.0	0.185	1.5	0.481	0.3	
*GNMT*	1.8	0.277	1.9	0.490	1.6	0.468	1.1	
*GOLGA1*	1.7	0.463	4.8	0.159	0.3	0.595	0.1	
*GOLGA3*	12.0	0.154	2.4	0.133	0	0.407	0.0	
*GSTZ1*	14.0	0.032	1.2	0.040	8.0	0.197	8.3	
*HINT1*	1.4	0.335	3.3	0.079	0.8	0.958	0.3	
*MAT1A*	4.6	0.028	8.8	0.040	1.8	0.475	0.3	
*NDUFA2*	3.2	0.045	6.0	0.046	1.3	0.619	0.3	
*OGDH*	2.0	0.355	2.0	0.545	2.0	0.407	1.3	
*PHB2*	1.0	0.817	0.7	0.461	1.6	0.495	2.8	
*PKLR*	3.5	0.069	2.3	0.373	7.0	0.141	3.8	
*RPL7A*	2.2	0.093	2.8	0.130	1.5	0.534	0.7	
*SDS*	9.0	0.026	1.6	0.014	1.0	0.879	0.8	
*SERBP1*	4.5	0.068	7.0	0.094	2.0	0.563	0.4	
*SUCLG2*	2.6	0.092	3.2	0.153	1.8	0.483	0.7	
*TLN1*	1.8	0.357	2.5	0.420	1.0	0.814	0.5	
*VWA8*	3.0	0.081	7.0	0.128	1.7	0.443	0.3	
*OTC*	2.1	0.319	2.3	0.606	2.0	0.334	1.1	Passed in the PCB-126-treated group
*KRT10*	1.0	0.876	0.4	0.371	3.3	0.272	9.3	
*LGALS9*	2.4	0.133	1.3	0.862	3.4	0.048	3.4	
*PCCA*	3.8	0.084	0.8	0.207	2.8	0.142	4.3	
*PRDX1*	2.2	0.182	2.8	0.391	1.8	0.353	0.8	
*SUCLG1*	2.0	0.179	1.6	0.633	2.7	0.111	2.1	
*TRIM21*	4.3	0.062	2.3	0.471	1.5	0.011	0.6	

Red color for a cell indicates a significant enrichment (*p* < 0.05) by FMO3 Co-IP in the Vehicle, PCB-126-treated, or in combined group.

**Table 2:** mRNA level of representative FMO3 PIPs in the FMO3 knockout mice and FMO3 overexpressed cells. Colors of the cell indicate trend and significance of change in the FMO3−/− mice or FMO3 overexpressed cells. The green color represents an increasing while the red color represents a decreasing change of this gene in the FMO3−/− mice or FMO3 overexpressed cells compared to their control counterparts. The deeper green/red indicates the change is significant (*p* < 0.05), while the lighter green/red indicates the trend of change can be observed but is not significant (0.05 < *p* < 0.1).

Gene Name	Function/ Compartment	FMO3−/−				Ms FMO3 Overexpression	
		C57BL/6 ♀		FVB ♀		AML12	
		Fold	*p*-Value	Fold	*p*-Value	Fold	*p*-Value
*Cps1*	Urea cycle	1.23	0.070	1.48	0.005	0.72	0.062
*Ass1*	Urea cycle	1.05	0.695	1.12	0.347	1.70	<0.001
*Arg1*	Urea cycle	1.41	0.008	1.28	0.008	0.71	0.221
*Otc*	Urea cycle	1.07	0.455	1.26	0.199	Not detected	
*Asl*	Urea cycle	1.26	0.018	1.50	0.001	0.78	<0.001
*Glul*	Ammonia metabolism	1.05	0.594	1.55	0.028	0.71	<0.001
*Vim*	Intermediate filament	1.52	0.027	1.53	0.034	1.26	0.001
*Krt18*	Intermediate filament	0.74	0.014	0.97	0.833	1.04	0.186
*Mvp*	Autophagic cell death	1.41	0.003	1.05	0.687	0.92	0.038
*Phb2*	Autophagic cell death	1.31	0.009	1.05	0.361	1.04	0.408
*C3*	Complement	0.99	0.910	1.19	0.350	0.75	0.036
*C1qb*	Complement	1.23	0.069	1.16	0.362	Not detected	
*Slc25a3*	Mitochondrial	1.41	0.030	1.19	0.187	0.80	<0.001
*Acaa2*	Mitochondrial	1.24	0.088	1.15	0.334	0.69	<0.001
*Dlst*	Mitochondrial	1.34	0.035	1.15	0.119	0.90	0.062
*Shmt1*	Mitochondrial	1.25	0.087	1.02	0.891	0.94	0.193

## Data Availability

Raw data files are available through a request to the corresponding author.

## Supplementary Materials

The following are available online at https://www.mdpi.com/article/10.3390/toxics10020060/s1, Figure S1: A. Western blot result shows PCB-126 treatment can induce expression of FMO3 in the male mouse liver (*n* = 5). B. Data show immunoprecipitation of FMO3 and its protein interaction partners (compared with normal IgG IP as background). C. Network model based on FMO3 protein-protein interaction partners identified in vehicle and PCB 126-treated groups. The inner circle protein identifications (nodes) are colored according to their relative enrichment to PCB126-treatment (red) or Vehicle (blue) and are ordered in a clockwise fashion relating to their relative enrichment. The outer circle proteins (grey) are known common interaction partners between FMO3 and its interaction partners or among multiple interaction partners of FMO3, Figure S2: The relative fold changes in transcription of 11 non-urea cycle-associated genes across the top biological processes (BP) and cellular components (CC) categories were compared between WT and FMO3-/- mouse livers on the female FVB [n=7(WT), 8(FMO3-/-)], Table S1: List of proteins detected more than half of the FMO3 IP samples, Table S2: Antibody list of western blotting, Table S3: List of primers for qPCR.
