# Supplementary material for: Proteomics-Based Identification of Interaction Partners of the Xenobiotic Detoxification Enzyme FMO3 Reveals Involvement in Urea Cycle
Source: Toxics. 2022 Jan 28;10(2):60. doi: 10.3390/toxics10020060 (PMC8877285; doi:10.3390/toxics10020060)
Supplement: Supplementary file 1 [file toxics-10-00060-s001.zip › toxics-1546894-supplementary.pdf]

toxics

MDPI

Supplementary Materials: Proteomics-Based Identification of Interaction Partners of the Xenobiotic Detoxification Enzyme FMO3 Reveals Involvement in Urea Cycle

Zhao Yang , Paul M. Stemmer and Michael C. Petriello

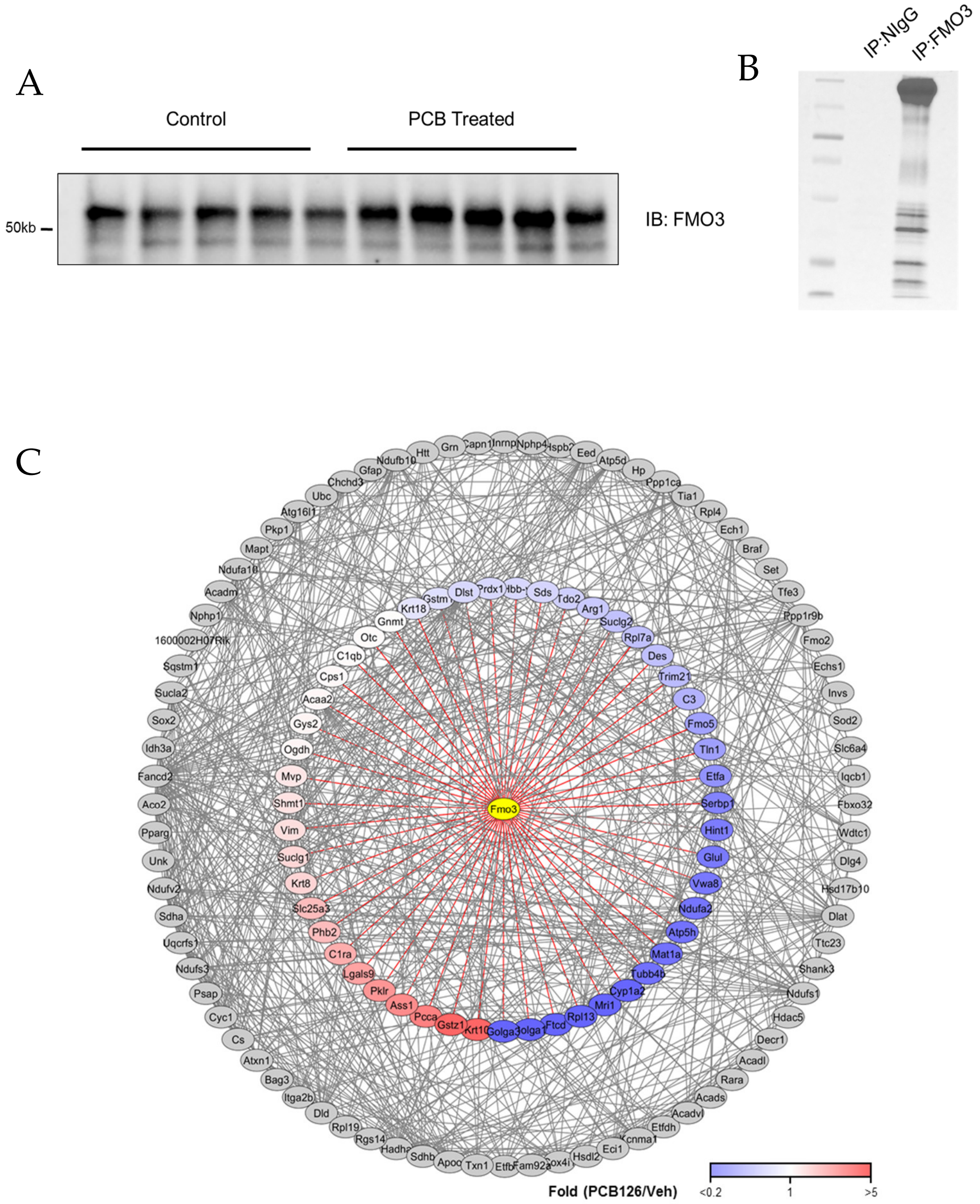

Figure S1. A. Western blot result shows PCB-126 treatment can induce expression of FMO3 in the male mouse liver (n=5). B. Data show immunoprecipitation of FMO3 and its protein interaction partners (compared with normal IgG IP as background). C. Network model based on FMO3 protein-protein interaction partners identified in vehicle and PCB 126-treated groups. The inner circle protein identifications (nodes) are colored according to their relative enrichment to PCB126-treatment (red) or Vehicle (blue) and are ordered in a clockwise fashion relating to their relative enrichment. The outer circle proteins (grey) are known common interaction partners between FMO3 and its interaction partners or among multiple interaction partners of FMO3.

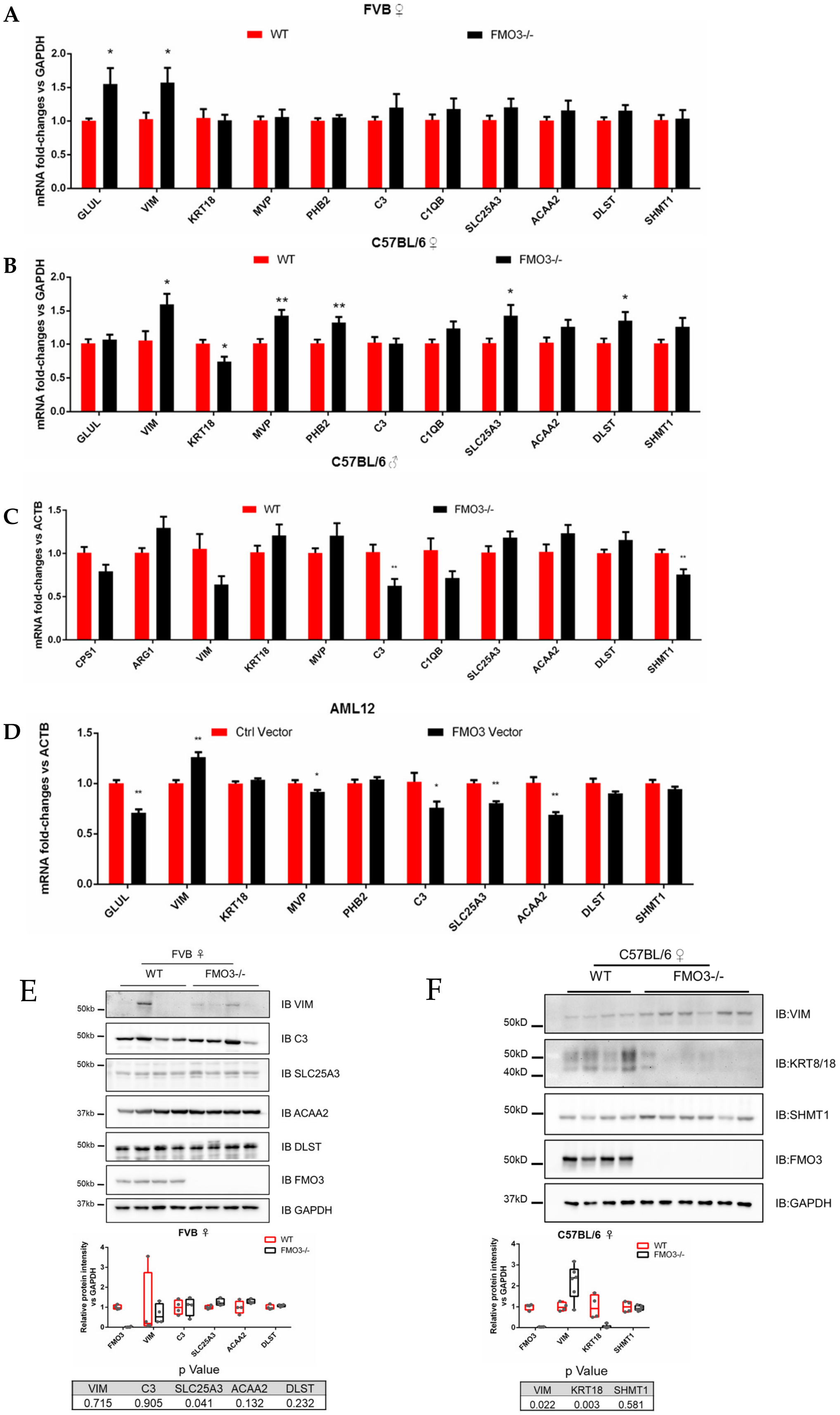

p Value

VIM	C3	SLC25A3	ACAA2	DLST
0.715	0.905	0.041	0.132	0.232

p Value

VIM	KRT18	SHMT1
0.022	0.003	0.581

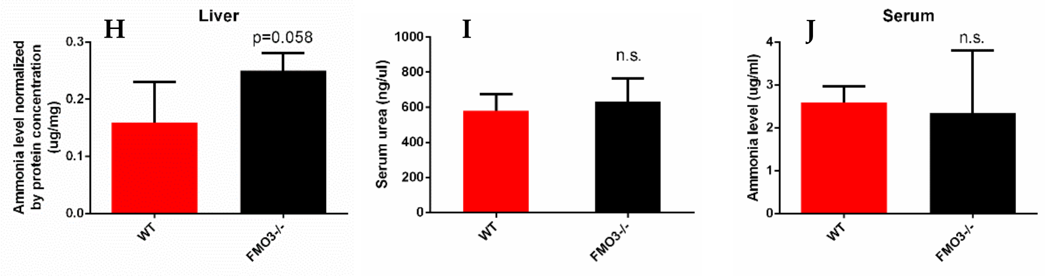

Figure S2. The relative fold changes in transcription of 11 non-urea cycle-associated genes across the top biological processes (BP) and cellular components(CC) categories were compared between WT and FMO3-/- mouse livers on the female FVB [n=7(WT), 8(FMO3-/-)](**A**) or C57BL6 background [n=7(WT), 6(FMO3-/-)] (**B**). Red bars represent gene expression in the WT while black bars represent FMO3-/- mouse results. *: p≤0.05; **:p≤0.01. **C.** The relative fold changes in transcription of 11 genes (including UCGs) across the top BP and CC categories were compared between WT and FMO3-/- mouse livers on the male C57BL6 background (n=6). Red bars represent gene expression in the WT while black bars represent FMO3-/- mouse results. *: p≤0.05; **:p≤0.01. **D.** The relative fold changes in transcription of 10 non-urea cycle-associated genes across the top BP and CC categories were compared between control or FMO3 overexpressed AML12 cells (n=4). Red bars represent gene expression in the control group while black bars represent FMO3 overexpressed results. *: p≤0.05; **:p≤0.01. Representative western blotting results of the genes previously changed at mRNA level between WT and FMO3-/- mouse livers on the female FVB (n=4) (**E**) or C57BL6 background [n=4(WT), 6(FMO3-/-)] (**F**). **G.** Representative western blotting results of the genes previously changed at mRNA level between control or FMO3 overexpressed AML12 cells (n=4). Average fold change of the FMO3 PIPs shown in the right panel has been quantified and normalized by the housekeeping gene GAPDH. P-values of each gene are listed below the quantification figures. **H.** Hepatic ammonia levels in the FVB FMO3-/- mice show increasing trend, but this trend is not significant (n=4, p=0.058). Serum urea (**I**) and ammonia (**J**) levels in the FVB female mice [n=4 (WT), 3(FMO3-/-)] show no significant change [p=0.643 (urea), 0.759 (ammonia)] between the two genotypes. Quantification of protein expression shown in **E** and **F** indicated 25 to 75 percentile as box and whiskers plot, and all individual values were indicated as the points. Otherwise data are means ± SEM.

Table S1. List of proteins detected by proteomics in more than half of the FMO3 IP samples.

#	Gene name	MW	Full name
1	CPS1	165 kDa	Carbamoyl-phosphate synthase [ammonia], mitochondrial
2	VIM	54 kDa	Vimentin
3	FMO3	61 kDa	Dimethylaniline monooxygenase [N-oxide-forming] 3
4	C3	186 kDa	Complement C3
5	ACAA2	42 kDa	3-ketoacyl-CoA thiolase, mitochondrial
6	ASS1	47 kDa	Argininosuccinate synthase
7	CYP1A2	58 kDa	Cytochrome P450 1A2
8	FMO5	60 kDa	Dimethylaniline monooxygenase [N-oxide-forming] 5
9	KRT8	55 kDa	Keratin, type II cytoskeletal 8
10	KRT18	48 kDa	Keratin, type I cytoskeletal 18
11	ACTA1	42 kDa	Actin, alpha skeletal muscle
12	TUBB4B	50 kDa	Tubulin beta-4B chain
13	DES	53 kDa	Desmin
14	MVP	96 kDa	Major vault protein
15	SHMT1	53 kDa	Serine hydroxymethyltransferase, cytosolic
16	DLST	49 kDa	Dihydrolipoyllysine-residue succinyltransferase component of 2-oxoglutarate dehydrogenase complex, mitochondrial
17	PRDX1	22 kDa	Peroxiredoxin-1

18	HBA	15 kDa	Hemoglobin subunit alpha
19	TUBB2A	50 kDa	Tubulin beta-2A chain
20	GSTM1	26 kDa	Glutathione S-transferase Mu 1
21	MRI1	39 kDa	Methylthioribose-1-phosphate isomerase
22	GLUL	42 kDa	Glutamine synthetase
23	PHB2	33 kDa	Prohibitin-2
24	GOLGA1	87 kDa	Golgin subfamily A member 1
25	ACLY	120 kDa	ATP-citrate synthase
26	KRT10	58 kDa	Keratin, type I cytoskeletal 10
27	FTCD	59 kDa	Formimidoyltransferase-cy-clodeaminase
28	C1QB	27 kDa	Complement C1q subcompo-nent subunit B
29	RPL13	24 kDa	60S ribosomal protein L13
30	OTC	40 kDa	Ornithine carbamoyltransferase, mitochondrial
31	TLN1	270 kDa	Talin-1
32	DBT	53 kDa	Lipoamide acyltransferase com-ponent of branched-chain alpha-keto acid dehydrogenase com-plex, mitochondrial
33	SLC25A3	40 kDa	Phosphate carrier protein, mito-chondrial
34	ETFA	35 kDa	Electron transfer flavoprotein subunit alpha, mitochondrial
35	C1RA	80 kDa	Complement C1r-A subcompo-nent
36	ARG1	35 kDa	Arginase-1
37	FMO2	61 kDa	Dimethylaniline monooxygen-ase [N-oxide-forming] 2
38	KRT5	62 kDa	Keratin, type II cytoskeletal 5
39	KRT17	48 kDa	Keratin, type I cytoskeletal 17
40	LGALS9	40 kDa	Galectin-9
41	MAT1A	44 kDa	S-adenosylmethionine synthase isoform type-1
42	SUCLG2	47 kDa	Succinate--CoA ligase [GDP-forming] subunit beta, mito-chondrial
43	OGDH	116 kDa	2-oxoglutarate dehydrogenase, mitochondrial
44	GNMT	33 kDa	Glycine N-methyltransferase
45	ATP5PD	19 kDa	ATP synthase subunit d, mito-chondrial
46	SORD	38 kDa	Sorbitol dehydrogenase
47	KRT2	71 kDa	Keratin, type II cytoskeletal 2 epidermal
48	SOD1	16 kDa	Superoxide dismutase [Cu-Zn]
49	ABCD3	75 kDa	ATP-binding cassette sub-family D member 3
50	RPL7A	30 kDa	60S ribosomal protein L7a
51	HINT1	14 kDa	Histidine triad nucleotide-bind-ing protein 1

52	ETFDH	68 kDa	Electron transfer flavoprotein-ubiquinone oxidoreductase, mitochondrial
53	GYS2	81 kDa	Glycogen [starch] synthase, liver
54	PCCA	80 kDa	Propionyl-CoA carboxylase alpha chain, mitochondrial
55	SCP2	59 kDa	Non-specific lipid-transfer protein
56	JCHAIN	18 kDa	Immunoglobulin J chain
57	TRIM21	54 kDa	E3 ubiquitin-protein ligase TRIM21
58	YWHAG	28 kDa	14-3-3 protein gamma
59	PKLR	62 kDa	Pyruvate kinase PKLR
60	RPL23A	18 kDa	60S ribosomal protein L23a
61	SUCLG1	36 kDa	Succinate--CoA ligase [ADP/GDP-forming] subunit alpha, mitochondrial
62	HBB-Y	16 kDa	Hemoglobin subunit epsilon-Y2
63	TDO2	48 kDa	Tryptophan 2,3-dioxygenase
64	GOLGA3	167 kDa	Golgin subfamily A member 3
65	RPL17	21 kDa	60S ribosomal protein L17
66	RPL27A	17 kDa	60S ribosomal protein L27a
67	DLAT	68 kDa	Dihydrolipoyllysine-residue acetyltransferase component of pyruvate dehydrogenase complex, mitochondrial
68	GSTZ1	24 kDa	Maleylacetoacetate isomerase
69	NDUFA2	11 kDa	NADH dehydrogenase [ubiquinone] 1 alpha subcomplex subunit 2
70	RPL11	20 kDa	60S ribosomal protein L11
71	ACACA	265 kDa	Acetyl-CoA carboxylase 1
72	SDS	35 kDa	L-serine dehydratase/L-threonine deaminase
73	AGXT	46 kDa	Serine--pyruvate aminotransferase, mitochondrial
74	GSTA3	25 kDa	Glutathione S-transferase A3
75	VWA8	213 kDa	von Willebrand factor A domain-containing protein 8
76	RPS16	16 kDa	40S ribosomal protein S16
77	RPS6	29 kDa	40S ribosomal protein S6
78	EPHX2	63 kDa	Bifunctional epoxide hydrolase 2
79	PON1	40 kDa	Serum paraoxonase/arylesterase 1
80	RPL7	31 kDa	60S ribosomal protein L7
81	ALS2	183 kDa	Alsin
82	RAI14	109 kDa	Ankycorbin
83	RPS26	13 kDa	40S ribosomal protein S26
84	RBMXL1	42 kDa	RNA binding motif protein, X-linked-like-1
85	SERBP1	45 kDa	Plasminogen activator inhibitor 1 RNA-binding protein
86	ACBD5	57 kDa	Acyl-CoA-binding domain-containing protein 5

87	TGM2	77 kDa	Protein-glutamine gamma-glutamyltransferase 2
88	UROC1	75 kDa	Urocanate hydratase
89	URAH	14 kDa	5-hydroxyisourate hydrolase
90	RPS20	13 kDa	40S ribosomal protein S20
91	RPS28	8 kDa	40S ribosomal protein S28
92	SARS	58 kDa	Serine--tRNA ligase, cytoplasmic
93	MCCC1	79 kDa	Methylcrotonoyl-CoA carboxylase subunit alpha, mitochondrial
94	FGG	49 kDa	Fibrinogen gamma chain
95	AP3B1	123 kDa	AP-3 complex subunit beta-1
96	SQSTM1	48 kDa	Sequestosome-1
97	HSD17B10	27 kDa	3-hydroxyacyl-CoA dehydrogenase type-2
98	RPL24	18 kDa	60S ribosomal protein L24
99	EHHADH	78 kDa	Peroxisomal bifunctional enzyme
100	SFXN1	36 kDa	Sideroflexin-1
101	ECI1	32 kDa	Enoyl-CoA delta isomerase 1, mitochondrial
102	RNH1	50 kDa	Ribonuclease inhibitor
103	TCOF1	135 kDa	Treacle protein
104	HIP1R	119 kDa	Huntingtin-interacting protein 1-related protein
105	TMEM205	21 kDa	Transmembrane protein 205
106	FGB	55 kDa	Fibrinogen beta chain
107	PCK1	69 kDa	Phosphoenolpyruvate carboxykinase, cytosolic [GTP]
108	PEX14	41 kDa	Peroxisomal membrane protein PEX14
109	RDX	69 kDa	Radixin
110	KIF5B	110 kDa	Kinesin-1 heavy chain
111	SNRPN	25 kDa	Small nuclear ribonucleoprotein-associated protein N
112	EEF1B	25 kDa	Elongation factor 1-beta
113	H2-L	41 kDa	H-2 class I histocompatibility antigen, L-D alpha chain
114	KHK	33 kDa	Ketohexokinase
115	ADK	40 kDa	Adenosine kinase

Table S2. Antibody list for western blotting.

Gene name	Company	Catalog #
FMO3	Abcam	ab126790
CPS1	Abcam	ab45956
ASL	Abcam	ab201026
ARG1	Cell Signaling	93668
ASS1	Cell Signaling	70720
VIM	Cell Signaling	5741
C3	Abcam	ab200999
SLC25A3	antibodies-online Inc.	ABIN5705036
ACAA2	Abcam	ab128911

DLST	Cell Signaling	12618
KRT8/18	Cell Signaling	4546
SHMT1	Cell Signaling	80715
GAPDH	Cell Signaling	5174

Table S3. List of primers for qPCR.

Gene name	Forward (5'->3')	Reverse (5'->3')
CPS1	CGGGAAGTAGAGATGGACGC	CCTTGGCTGATGGTCTGTGT
ASS1	TGGGGCCAAAAAGGTGTTCA	ACATACTTGGCCCCTTCACG
ARG1	GTACATTGGCTTGCGAGACG	ATCGGCCTTTTCTTCCTTCCC
OTC	TTAGTGTTCCCAGAGGCAGAG	CTGGAGCACAGGTGAGTAGT
ASL	CAGAGGCGGAACGTGATGTC	GAATCTCGTGTCAGCGCAAC
GLUL	GGGTATGCATGTCCGGTAGG	GGGCCTGCCCTTTCAACTAT
VIM	TTCTCTGGCACGTCTTGACC	GCTTGGAAACGTCCACATCG
KRT18	GCCAGGCCCAGGAATATGAA	AGGGCATCGTTGAGACTGAAA
MVP	TAGCCATTGAGACGGAGGCT	AGCCAAGTCCCTGATGGTTC
PHB2	CGTGGAAGGCGGTCATAGAG	GGGGATCCTGAAGTGAAGGC
C3	CCCAATGTCCTACGGCTGG	TCCTTACTGGCTGGAATCTTGA
C1QB	GATAAAGGGGGAGAAAGGGCT	GGACCCTTAGGGCCAACTTT
SLC25A3	CCGCCGTGGAAGAGTACAGT	TTAAGACCCCACCAAAGCCAC
ACAA2	ACGTGAACGAAGCTTTTGCC	TTCCACCTCGACGCCTTAAC
DLST	GCCCGGAAGAATGAACTTGC	TCGAACTTCCACCTTGCCTC
SHMT1	CTCAGTGCTGGGGTGTCAAT	AGGTCCAAGCCCATGATTCG
FMO3	ACTGGTGGTACACAAGGCAG	GCTCATCATTGAACACGGGC
ACTB	GCCACTGTCGAGTCGCGT	GATACCTCTCTTGCTCTGGGC
GAPDH	CAAGGAGTAAGAAACCCTGGACC	CGAGTTGGGATAGGGCCTCT
